# Dissemination of Cost Action FP0905 information by website

**DOI:** 10.1186/1753-6561-5-S7-O60

**Published:** 2011-09-13

**Authors:** Fabio Migliacci, Matthias Fladung, Cristina Vettori, Jean-Charles Leplé, Klaus Minol, Francesca Donnarumma

**Affiliations:** 1 Organo Metallic Chemistry Compound Institute, CNR, Via Madonna del Piano 10, I-50019 Sesto Fiorentino (FI), Italy; 2 Johann Heinrich von Thuenen Institute (vTI), Institute of Forest Genetics, Sieker Landstr. 2, D-22927 Grosshansdorf, Germany; 3 Plant Genetics Institute, CNR, Via Madonna del Piano 10, I-50019 Sesto Fiorentino (FI), Italy; 4 INRA, UR588 AGPF, Centre de recherche d'Orléans, 2163 Avenue de la Pomme de Pin, CS 40001 Ardon, 45075 Orléans Cedex 2, France; 5 Genius GmbH, Robert-Bosch-Str. 7, 64293 Darmstadt, Germany

## Background

The Cost Action FP0905 aims to evaluate the scientific knowledge of GMT biosafety protocols coordinating the existing information generated in various European countries as basis for future EU policy and regulation for the environmental impact assessment. For spreading the information relevant to the Action, the website http://www.cost-action-fp0905.eu/has been set up, educating the general population of the technical, social, environmental and economic aspects of this issue. The principal aims of the website are: to provide a database with the main information on forest GMTs, that should be available to the scientific community and Europe organisations; to update the website with science-based information for public interest in the utilization of GMTs in forest plantation and at the same time safeguarding the environment, using all the information collected to identify particular topics which could be useful to develop research projects collaborating with the other working groups.

## Material and methods

The Website was built using the Open Source Software Joomla, a program for web site design and content management. This software has not been written for profit, but to benefit the user community. The difference between Joomla and other web design software systems is that there are thousands of fully functional, complete web site templates available in the Internet, so that all what is to do is to choose the layout you like and to add information in the templates.

However, Joomla as a "Content Management System" is not only a “simple” web site construction program, furthermore it can also be used as a database. It is used to build/design a “php/mysql database” to collect the information. But the possibilities of the program on how to use it as a database are endless. In this way it will be simple to create the database tables and fields. The software generates the scripts to upload them into the website, to setup the MySQL tables and the scripts and to manage the tables once uploaded.

The most important features of the Joomla program are:

1. Instantly publish PHP MySQL database applications ready for uploading on the website of Action.

2. Manage easily the database.

3. Automatic generation of php forms to display and browse data content of tables, with the ability to edit, delete and add records.

4. Data navigation, sorting, searching, search filtering, modification, addition, deletion, file uploads. The website is also a very friendly searching engine. Google will regularly visit this website site, due to the frequent content changes that result from the news feeds. They are usually updated on a daily basis, even if the web site is not updated on a regular basis.

## Results

Working group 4 is developing and managing the web site of the Action enhancing communication and information among the participants and with those organisations interested in the data provided. The website has provided a forum for the organizations working within and outside the consortium; discussing for example, any effect of a transgenic plantation needs in the context of already existing and accepted forestry practise. The Action has also been introduced in facebook.

**Figure 1:**
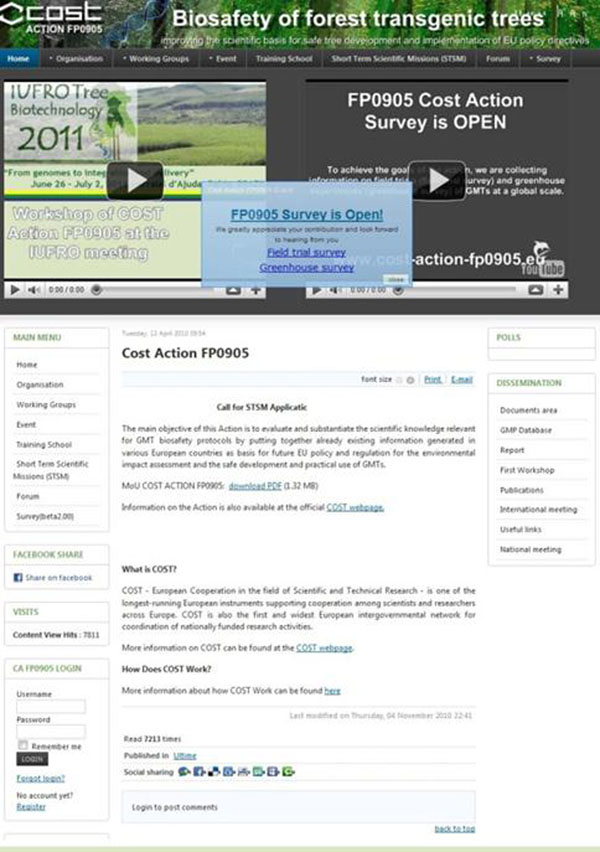
image of the COST Action FP0905 website

